# Long-term functional outcomes and quality of life after partial glossectomy for T2 squamous cell carcinomas

**DOI:** 10.1016/j.bjorl.2021.06.009

**Published:** 2021-07-30

**Authors:** Giuseppe Riva, Silvia Sapino, Mattia Ravera, Giulia Elia, Giancarlo Pecorari

**Affiliations:** University of Turin, Department of Surgical Sciences, Division of Otorhinolaryngology, Turin, Italy

**Keywords:** Oral cancer, Partial glossectomy, Flap reconstruction, Swallowing, Tongue motility

## Abstract

**Introduction:**

Partial glossectomy and reconstruction strategy for malignant tongue tumors influences speech and swallowing.

**Objective:**

The aim of this retrospective study was to evaluate long-term functional outcomes after partial glossectomy for pT2 mobile tongue carcinomas with a maximum dimension between 2 and 3 cm. Different reconstruction strategies (with or without pedicled flap) were compared.

**Methods:**

Twenty-two patients with at least 12 months followup were included. Clinician-based and self-reported instruments were used to analyze tongue motility, speech intelligibility and articulation, swallowing, and quality of life.

**Results:**

Patients with a higher tongue motility had better articulation and lower dysphagia. Avoiding pedicled flap reconstruction seemed to guarantee lower impairment of speech and swallowing. Worse functional outcomes induced a lower quality of life.

**Conclusion:**

Partial glossectomy results in tongue motility impairment and consequently alterations of oral functions. Since the type of reconstruction impacts long-term outcomes, it should be adequately planned before surgery.

## Highlights

• Partial glossectomy and reconstruction strategy influences speech and swallowing.
• Patients with a higher tongue motility had better articulation and less dysphagia.
• Worse functional outcomes induced a lower quality of life.

## Introduction

Oral cancer is the sixth most widespread worldwide tumor, representing 2.1% of all tumors, with 300,000 new cases and 145,000 death per year.^1^ The oral cavity plays an important role for the essential functions of speech and swallowing. Moreover, it is involved in emotional expression and social interaction. Treatments for oral cancer negatively impact on these functions. In particular, surgery for tumors of the mobile tongue can decrease speech and swallowing functions, thus reducing patients’ Quality of Life (QoL).^2^

In the last decades, the development of tongue reconstruction with pedicled or free flaps allowed better functional results, especially for resectable, locally advanced cancer. As a consequence, QoL improved.^2^ Besides survival, functional outcomes and QoL have become an essential part of the therapeutic path. The mobile tongue has a number of functions, including articulation, mastication, deglutition, and taste. Surgical defects mainly affect swallowing and speech articulation. In particular, patients may report bolus preparation deficit, delay in starting swallowing, oral remnants after swallowing, alterations in word articulation with phonemes distortion.^3^, ^4^, ^5^, ^6^

Functional outcomes are affected by extension and localization of tongue resection, type of reconstruction, motility of residual tongue, and adjuvant Radiotherapy (RT).^3^, ^4^, ^5^, ^6^ Type of reconstruction is usually chosen basing on extension of the tumor and subsequent resection.^7^ In particular, wedge-shaped tongue resection (usually less than one quarter of the mobile tongue) for small tumors (T1) are followed by primary or secondary intention closure, while hemiglossectomy (usually performed for tumors >3 cm) requires flap reconstruction.^7^ Intermediate tumors with a diameter between 2 and 3 cm (staged as T2 according to pathologic Tumor Nodes Metastasis – pTNM – staging system, VI edition) are generally treated with partial glossectomy (i.e., resection of less than half of the mobile tongue) and sometimes flap reconstruction. Contrasting data are present in the literature about functional outcomes after partial glossectomy with primary closure or flap reconstruction.^8^, ^9^, ^10^, ^11^ Indeed, flaps could add tissue bulk to the remnant tongue favoring swallowing, but may limit tongue movements impairing speech.^8^ The main limit of previous studies is the absence of samples’ homogeneity. In particular, different resections were analyzed all together and were performed in patients with different tumor stages.

The aim of this retrospective study was to evaluate long-term functional outcomes in patients who underwent partial glossectomy for pT2 tongue carcinomas, whose maximum dimensions ranged between 2 and 3 cm. We included partial resection of 25%–50% of the mobile tongue in order to analyze a homogeneous sample. We assessed tongue motility and its relationship with swallowing and speech functions. Moreover, quality of life was evaluated. Finally, we compared functional outcomes of two different reconstruction strategies (with or without pedicled flap).

## Methods

The study sample was composed of 22 out of 42 patients consecutively treated for pT2 squamous cell carcinoma of the mobile tongue (according to pathologic Tumor Nodes Metastasis – pTNM – staging system, VIII edition) at our department between 2011 and 2016. Patients with maximum tumor dimension between 2 and 3 cm were included in the study. According to TNM staging system, VIII edition, depth of invasion was between 5 and 10 mm. All the patients underwent partial glossectomy (i.e., resection of less than half of the mobile tongue, but more than one quarter) and ipsilateral selective neck dissection. Exclusion criteria were: surgery for relapse or head and neck second tumor; involvement of median tongue line; laryngeal, esophageal or neurological disorders that alter swallowing or phonation; previous head and neck radiation therapy; pT2 tumor with maximum dimension between 3 and 4 cm; follow-up <12 months. Twenty out of 42 patients were excluded because of relapse or death during followup or presence of exclusion criteria. We performed a chart review collecting clinical data (age, sex, history of smoking, alcohol consumption, pTNM stage, tumor dimension, adjuvant treatment, tongue motility, swallowing and articulation outcomes, quality of life). Long-term evaluation of functional outcomes was performed at a mean followup of 29.27 ± 19.52 months (range 12–70 months). The patients were divided in two groups according to reconstruction strategy (based on surgeons’ choice): without flap (Group A, 8 patients) or with pedicled flap reconstruction (Group B, 14 patients). All the patients underwent postoperative speech therapy. All procedures were in accordance with the ethical standards of the institutional research committee and with the World Medical Association Declaration of Helsinki (version 2002) and its later amendments or comparable ethical standards. Approval by the Institutional Review Board was not needed because of the retrospective nature of the study. Written informed consent was obtained from all participants included in the study.

The Radiation Therapy Oncology Group (RTOG) – European Organization for Research and Treatment of Cancer (EORTC) late radiation morbidity scoring system was used to assess long-term side effects of radiation therapy.^12^ It is a 5 point scale, from 0 (normal tissue) to 5 (radiation-induced necrosis). We considered the items concerning skin, subcutaneous tissue, mucosa, salivary gland, and larynx. The grading of dysphagia was based on the Common Terminology Criteria for Adverse Events version 4.0 (CTCAE, grade 0 = none; grade 1 = symptomatic, able to eat regular diet; grade 2 = symptomatic and altered eating/swallowing; grade 3 = severely altered eating/swallowing, tube feeding or total parenteral nutrition or hospitalization indicated; grade 4 = life-threatening consequences, urgent intervention indicated).^13^ We used this scale not only for irradiated patients, but also for those who did not undergo adjuvant therapy, in order to obtain a common assessment of swallowing disorders.

The tongue motility assessment (TMA) consisted of the evaluation of 9 tongue positions with a 3-point scale (1, marked impairment; 2, mild impairment; 3, normal).^4^, ^14^ The analyzed positions of the tongue were as follows: (1) Protrusion: “Stick your tongue out as far as possible”, (2) Protrusion and elevation: “Bring your tongue tip up towards the tip of your nose”, (3) Protrusion and depression: “Bring the tongue tip down towards your chin”, (4) Protrusion and left lateralization: “Bring your tongue to the left corner of your mouth”, (5) Protrusion and right lateralization: “Bring your tongue to the right corner of your mouth”, (6) Elevation: “Bring your tongue up to the hard palate”, (7) Retroflexion: “Bring your tongue tip as far back in your mouth as you can”, (8) Dorsal elevation: “Bring up the back of your tongue as if saying ‘k”, (9) Retraction: “Pull your tongue back in your mouth as far as possible”. A score was assigned to each tongue position. A mean total score was calculated for each patient. The evaluations were performed by the same physician for all the patients.

The speech intelligibility was scored on a scale ranging from 1 to 7 (1 – unintelligible, 2 – inadequate, 3 – difficult to understand, 4 – intelligible with careful listening, 5 – intelligible but with noticeable errors, 6 – occasional errors, 7 – no errors in continuous speech).^15^ A similar 7-point scale was used to evaluate subjects’ speech articulation (1 – unintelligible, 2 – inadequate, not acceptable, 3 – marginally acceptable, 4 – distorted but improved with multiple repetition, 5 – distorted but acceptable, 6 – occasional errors, 7 – no errors).^16^ Speech intellegibility and articulation were evaluated based on the conversation with the patient.^8^

Two questionnaires were self-administered to evaluate functional outcomes. In particular, Speech Handicap Index was used to assess speech problems, while M.D. Anderson Dysphagia Inventory to evaluate swallowing disorders.^17^, ^18^, ^19^, ^20^ The SHI questionnaire consists of 30 items based on a 5-point scale (“never”, “almost never”, “sometimes”, “almost always”, and “always”). A final question investigates the overall speech quality item, based on a 4-point scale (“good,” “reasonable,” “poor,” and “severe”). A total SHI score is calculated by summing all items (range 0–120). Two subscales, each with 14 items, evaluate psychosocial and speech functions, respectively. Higher scores indicate higher levels of speech-related problems. The MDADI questionnaire is composed by 20 items based on a 5-point scale (“strongly agree”, “agree”, “no opinion”, “disagree”, and “strongly disagree”). A total MDADI score is calculated by inverting the scores of two items (5 and 15) and then summing all items (range 0–100). Four subscales evaluate global, emotional, physical, and functional perceptions of swallowing ability (1, 6, 8, and 5-items, respectively). MDADI subscale scores are normalized to range from 20 (extremely low-functioning) to 100 (high-functioning).

The patients’ quality of life was evaluated with the European Organization for Research and Treatment of Cancer Quality-of-Life-Questionnaire-C30 (EORTC QLQ-C30) and the European Organization for Research and Treatment of Cancer Quality-of-Life Questionnaire-Head and Neck 35 (EORTC QLQ-H&N35).^21^ The EORTC QLQ-C30 includes a global score, 5 functional scales, and 9 symptoms scales. Higher scores for symptomatic scales indicate severe symptoms, while higher scores for the global QoL and the functional scales suggest a better level of functioning. The EORTC QLQ-H&N35 is a specific questionnaire for patients with head and neck cancer. It evaluates symptoms severity and is divided into 17 scales. Higher scores indicate more severe symptoms. All the protocols used in this study were validated for Italian language.

All statistical analyses were carried out using the Statistical Package for Social Sciences, version 20.0 (IBM Corp., Armonk, NY, USA). A descriptive analysis of all data was performed, and they were reported as means or percentages and standard deviations. The Kolmogorov–Smirnov test demonstrated a non-Gaussian distribution of variables, so non-parametric tests were used. The Mann–Whitney *U* test was used to assess differences between two independent groups in the mean of continuous variables. The Chi-Squared test was used for categorical variables. Spearman’s test was used to assess the correlations between continuous variables. A *p* < 0.05 was considered statistically significant.

## Results

### Patients

Mean age of the study sample (22 patients) was 65.00 ± 15.62 years (range 38–91 years). Mean tumor dimension was 2.39 ± 0.30 cm (range 2–3 cm). Table 1 reports clinical characteristics of the whole sample and the two groups.

**Table 1:** Patients and tumor characteristics.

Characteristics	Whole sample (n = 22)	Group A (n = 8)	Group B (n = 14)	*p*-Values^a^
Mean age (years)	65.00 ± 15.62	77.25 ± 12.80	58.00 ± 12.68	0.005
Sex (n, %)				0.225
Male	12 (54.5)	3 (37.5)	9 (64.3)	
Female	10 (45.5)	5 (62.5)	5 (35.7)	
Smoker (n, %)				
Pre-operative	14 (63.6)	5 (62.5)	9 (64.3)	0.321
Nowadays	2 (9.1)	1 (12.5)	1 (7.1)	0.127
Alcohol consumption (n, %)				
Pre-operative	9 (40.9)	3 (37.5)	6 (42.9)	0.412
Nowadays	6 (27.3)	2 (25.0)	4 (28.6)	0.385
T (pTNM VIII ed.) (n, %)				1.000
T1	0 (0)	0 (0)	0 (0)	
T2	22 (100)	8 (100)	14 (100)	
T3	0 (0)	0 (0)	0 (0)	
T4	0 (0)	0 (0)	0 (0)	
N (pTNM VIII ed.) (n, %)				0.693
N0	19 (86.4)	7 (87.5)	12 (85.8)	
N1	2 (9.1)	1 (12.5)	1 (7.1)	
N2	1 (4.5)	0 (0)	1 (7.1)	
N3	0 (0)	0 (0)	0 (0)	
M (pTNM VIII ed.) (n, %)				1.000
M0	22 (100)	8 (100)	14 (100)	
M1	0 (0)	0 (0)	0 (0)	
Stage (n, %)				0.439
I	0 (0)	0 (0)	0 (0)	
II	21 (95.5)	8 (100)	13 (92.9)	
III	1 (4.5)	0 (0)	1 (7.1)	
IVa	0 (0)	0 (0)	0 (0)	
IVb	0 (0)	0 (0)	0 (0)	
Grading (n, %)				0.387
G1	0 (0)	0 (0)	0 (0)	
G2	17 (77.3)	7 (87.5)	10 (71.4)	
G3	5 (22.7)	1 (12.5)	4 (28.6)	
Maximum tumor dimension (cm)	2.39 ± 0.30	2.38 ± 0.23	2.41 ± 0.34	1.000
Follow-up (months)	29.27 ± 19.52	25.37 ± 16.83	31.50 ± 21.17	0.664

pTNM, pathological tumor node metastasis staging system.

a Comparison between group A and B.

Surgical treatments are highlighted in Table 2. Postoperative speech and swallowing rehabilitation therapy was administered in all the cases. No patient had tracheostomy and/or enteral nutrition at the time of evaluation. Ten patients underwent adjuvant treatments. The RTOG/EORTC late radiation morbidity scoring system showed that 80% of irradiated patients had mild to moderate toxicity (grade 1–2) regarding skin, subcutaneous tissue and mucosa, while 40% of cases had grade 1–2 salivary and laryngeal toxicity. The other irradiated subjects had no radiotherapy toxicities. The CTCAE scale for dysphagia showed a mild-moderate impairment (grade 1–2) in 14 cases (63.6%), while 8 patients (36.4%) did not report any swallowing complaints.

**Table 2:** Treatment characteristics of the sample (n = 22).

Reconstruction	n (%)
Without flap (Group A)	8 (36.4)
Primary closure	6 (27.3)
Partial closure	2 (9.1)
With flap (Group B)	14 (63.6)
Platysma myocutaneous flap	8 (36.3)
Pectoralis major myofascial flap	4 (18.2)
Pectoralis major myocutaneous flap	2 (9.1)

**Figure 1:**
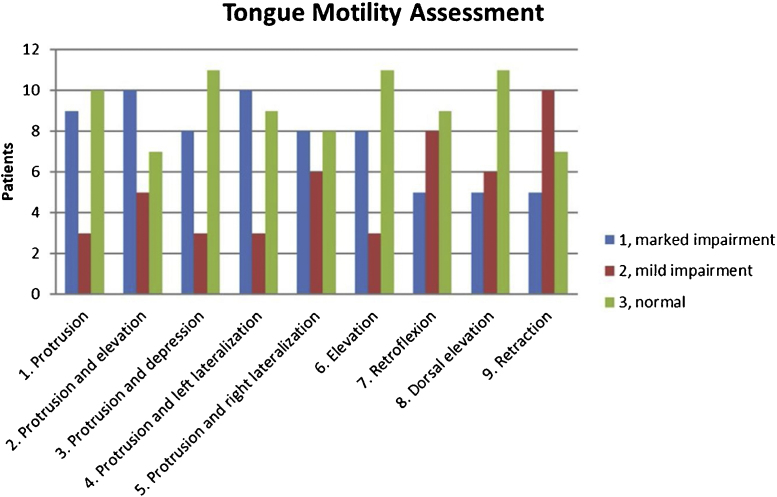
Tongue motility assessment of the whole sample (22 patients), with results for each tongue movement.

Mean TMA score was 2.08 ± 0.79 (range 1–3). Better outcomes (50% of patients with a score of 3) were observed for the following three tongue movements: protrusion with depression, elevation, and dorsal elevation. On the contrary, higher impairment (45.5% of cases with a score of 1) was reported for the following two movements: protrusion with elevation, and protrusion with lateralization (Fig. 1).

Speech intelligibility score was good, with 72.8% of patients having a score ≥6. The articulation score was slightly lower than intelligibility, with 77.3% of subjects having a score of 5 or 6 (Table 3). Concerning SHI, 90.9% of the patients reported a good or reasonable speech, with a mean total score of 37.18 ± 29.21, suggesting a good subjective evaluation of speech (Table 4). MDADI showed high values for emotional and functional scales, while mean global score was slightly lower (65.45 ± 32.76) (Table 4). Mean MDADI total score was 72.31 ± 23.97, indicating good functioning. Global QoL at EORTC QLQ-C30 questionnaire was good (mean score 69,32 ± 13,70) (Table 5).

**Table 3:** Speech intelligibility and articulation scores (n, %).

Scores	Whole sample (n = 22)	Group A (n = 8)	Group B (n = 14)	*p*-Values^a^
Intelligibility				0.502
1 – unintelligible	0 (0)	0 (0)	0 (0)	
2 – inadequate	0 (0)	0 (0)	0 (0)	
3 – difficult to understand	1 (4.5)	0 (0)	1 (7.1)	
4 – intelligible with careful listening	2 (9.1)	0 (0)	2 (14.3)	
5 – intelligible but with noticeable errors	3 (13.6)	1 (12.5)	2 (14.3)	
6 – occasional errors	8 (36.4)	4 (50.0)	4 (28.6)	
7 – no errors in continuous speech	8 (36.4)	3 (37.5)	5 (35.7)	
Articulation				0.146
1 – unintelligible	0 (0)	0 (0)	0 (0)	
2 – inadequate, not acceptable	0 (0)	0 (0)	0 (0)	
3 – marginally acceptable	2 (9.1)	0 (0)	2 (14.3)	
4 – distorted but improved with multiple repetition	3 (13.6)	0 (0)	3 (21.4)	
5 – distorted but acceptable	9 (40.9)	4 (50.0)	5 (35.7)	
6 – occasional errors	8 (36.4)	4 (50.0)	4 (28.6)	
7 – no errors	0 (0)	0 (0)	0 (0)	

a Comparison between group A and B.

**Table 4:** Speech Handicap Index and M.D. Anderson dysphagia inventory scores.

Scores	Whole sample (n = 22)	Group A (n = 8)	Group B (n = 14)	*p*-Values^a^
Speech Handicap Index				
Overall speech quality item (n, %)				0.668
Good	0 (0)	0 (0)	0 (0)	
Reasonable	9 (40.9)	4 (50.0)	5 (35.7)	
Poor	11 (50.0)	3 (37.5)	8 (57.1)	
Severe	2 (9.1)	1 (12.5)	1 (7.2)	
Psychosocial subscale (mean ± standard dev.)	14.23 ± 14.94	10.75 ± 16.76	16.21 ± 14.06	0.423
Speech subscale (mean ± standard dev.)	20.64 ± 13.56	15.25 ± 15.89	23.71 ± 11.54	0.164
SHI total score (mean ± standard dev.)	37.18 ± 29.21	27.63 ± 33.03	42.64 ± 27.73	0.246
M.D. Anderson dysphagia inventory				
Global scale (mean ± standard dev.)	65.45 ± 32.76	82.50 ± 29.15	55.71 ± 31.55	0.061
Emotional subscale (mean ± standard dev.)	72.57 ± 25.22	81.24 ± 24.75	67.62 ± 24.99	0.216
Physical subscale (mean ± standard dev.)	70.11 ± 25.44	82.81 ± 21.65	62.85 ± 25.24	0.092
Functional subscale (mean ± standard dev.)	76.91 ± 25.18	85.50 ± 22.82	72.00 ± 24.35	0.190
MDADI Total score (mean ± standard dev.)	72.31 ± 23.97	83.00 ± 22.29	66.21 ± 23.46	0.110

a Comparison between group A and B.

**Table 5:** EORTC QLQ-C30 and H&N35 scores (mean ± standard deviation).

Scores	Whole sample (n = 22)	Group A (n = 8)	Group B (n = 14)	*p*-Values*
**EORTC QLQ-C30**				

***Global Health Status/QoL***	69.32 ± 13.70	70.83 ± 11.78	68.45 ± 15.04	0.571

***Functional scales***				
Physical functioning	82.42 ± 19.47	84.16 ± 19.17	81.43 ± 20.28	0.677
Role functioning	84.09 ± 26.47	85.42 ± 24.29	83.33 ± 28.49	0.878
Emotional functioning	76.89 ± 21.04	87.50 ± 14.08	70.83 ± 22.35	0.072
Cognitive functioning	90.15 ± 17.56	83.33 ± 21.82	94.04 ± 14.03	0.071
Social functioning	83.33 ± 23.00	93.75 ± 12.40	77.38 ± 25.83	0.083

***Symptom scales***				
Fatigue	26.26 ± 25.32	31.94 ± 30.53	23.02 ± 22.42	0.505
Nausea and vomiting	4.54 ± 9.17	8.33 ± 12.59	2.38 ± 6.05	0.191
Pain	14.39 ± 19.45	12.50 ± 23.14	15.47 ± 17.85	0.524
Dyspnea	16.66 ± 24.66	16.66 ± 17.81	16.66 ± 28.49	0.636
Insomnia	24.24 ± 32.82	20.83 ± 35.35	26.19 ± 32.49	0.597
Appetite loss	12.12 ± 26.32	12.50 ± 35.35	11.90 ± 21.11	0.514
Constipation	21.21 ± 36.43	16.66 ± 35.63	23.81 ± 37.96	0.619
Diarrhea	0.00 ± 0.00	0.00 ± 0.00	0.00 ± 0.00	1.000
Financial difficulties	12.12 ± 28.26	0.00 ± 0.00	19.04 ± 33.87	0.104

**H&N35**				

***Symptom scales***				
Pain	18.18 ± 16.99	10.41 ± 15.26	22.62 ± 16.80	0.101
Swallowing	18.56 ± 18.71	14.58 ± 17.10	20.83 ± 19.81	0.413
Senses problems	18.18 ± 26.68	14.58 ± 24.29	20.23 ± 28.62	0.701
Speech problems	22.73 ± 23.12	16.66 ± 22.22	26.18 ± 23.71	0.328
Trouble with social eating	17.80 ± 24.84	11.45 ± 22.24	21.42 ± 26.29	0.141
Trouble with social contact	10.00 ± 13.76	6.66 ± 13.80	11.90 ± 13.88	0.323
Less sexuality	37.88 ± 34.95	41.66 ± 42.72	35.71 ± 31.25	0.860
Teeth	21.21 ± 31.78	8.33 ± 15.42	28.57 ± 63.64	0.187
Opening mouth	25.75 ± 27.08	16.66 ± 25.19	30.95 ± 27.62	0.226
Dry mouth	30.30 ± 32.38	37.50 ± 37.53	26.18 ± 29.75	0.490
Sticky saliva	30.30 ± 36.95	20.83 ± 35.35	35.71 ± 38.03	0.268
Coughing	16.66 ± 22.42	20.83 ± 24.80	14.28 ± 21.53	0.506
Felt ill	6.06 ± 13.15	8.33 ± 15.42	4.76 ± 12.10	0.540
Pain killers	18.18 ± 39.48	12.50 ± 35.35	21.42 ± 42.58	0.610
Nutritional supplements feeding tube	27.27 ± 45.58	25.40 ± 46.29	28.57 ± 46.88	0.860
Feeding tube	0.00 ± 0.00	0.00 ± 0.00	0.00 ± 0.00	1.000
Weight loss	13.63 ± 35.12	12.50 ± 35.00	14.28 ± 36.31	0.909
Weight gain	18.18 ± 39.47	25.00 ± 46.29	14.28 ± 36.31	0.540

* Comparison between group A and B.

### Correlations among functional outcomes

Considering the whole sample (22 patients), mean TMA score had a statistically significant correlation with articulation score (*p* = 0.032) and CTCAE dysphagia score (*p* = 0.004), being TMA higher if the subject had a better articulation or a lower dysphagia. Moreover, mean TMA score correlated to SHI total score (Spearman’s Rho = −0.583, *p* = 0.004) and MDADI total score (Spearman’s Rho = 0.643, *p* = 0.001). A near significant correlation was observed between mean TMA score and EORTC QLQ-C30 Global Health Status/QoL (Spearman’s Rho = 0.418, *p* = 0.053).

CTCAE dysphagia scale correlated with MDADI total score (*p* = 0.001), while articulation score related to SHI total score (*p* = 0.035). A statistically significant correlation was seen between SHI total score and MDADI total score (Spearman’s Rho = −0.919, *p* < 0.001), and between these two parameters and EORTC QLQ-C30 Global Health Status/QoL (Spearman’s Rho = −0.752, *p* < 0.001, and Spearman’s Rho = 0.646, *p* = 0.001, respectively).

**Figure 2:**
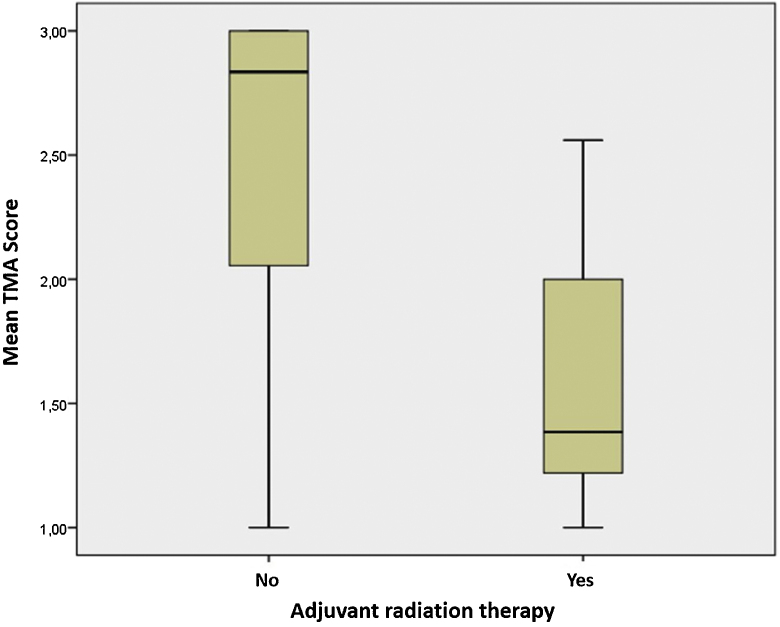
Impact of adjuvant radiation therapy on tongue motility.

Finally, adjuvant radiation therapy had a negative impact on tongue motility: mean TMA score was 2.48 ± 0.74 in non-irradiated patients and 1.59 ± 0.57 in irradiated ones (*p* = 0.009) (Fig. 2). No significant correlations were observed between RT and other outcomes, such as articulation score, CTCAE dysphagia scale, SHI total score, MDADI total score, and EORTC QLQ-C30 Global Health Status/QoL (*p* = 0.786, 0.346, 0.276, 0.418, 0.674, respectively). However, a trend in favor of non-irradiated subjects was present for these variables.

### Comparison between reconstruction strategies

Concerning clinical characteristics, only age showed a statistically significant difference between the two different reconstruction strategies (Table 1). In particular, patients with pedicled flap reconstruction were younger than others (*p* = 0.005). Adjuvant radiation therapy was performed in 3 patients (37.5%) in group A and 7 (50.0%) in group B (*p* > 0.05) No statistically significant differences between the two subgroups about radiotherapy side effects were observed (*p* > 0.05). Assessment of dysphagia by CTCAE showed a higher percentage of mild-moderate impairment (grade 1–2) in group B (71.4%), compared to group A (50%) (*p* = 0.02).

**Figure 3:**
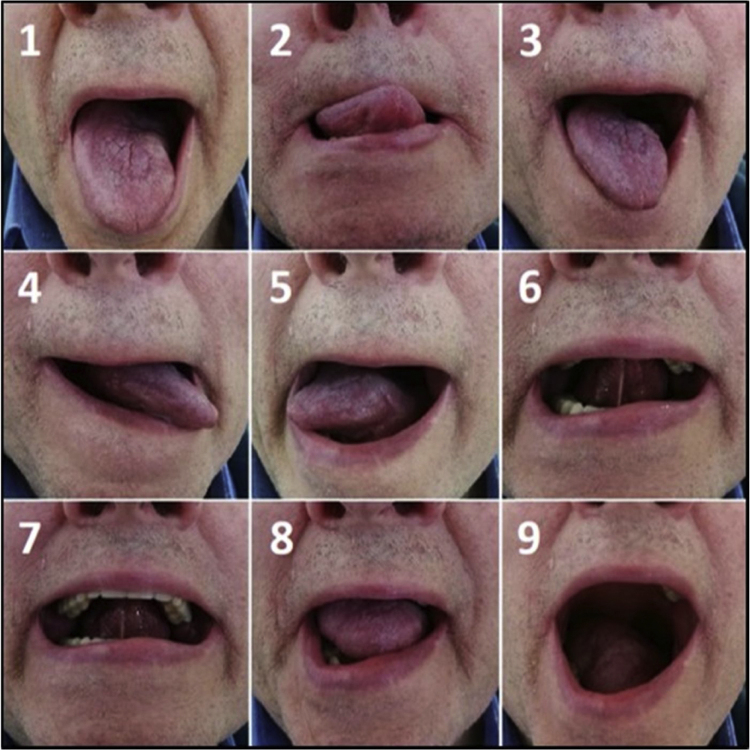
The nine movements of the tongue motility assessment in patient who underwent left partial glossectomy without flap reconstruction (primary closure).

**Figure 4:**
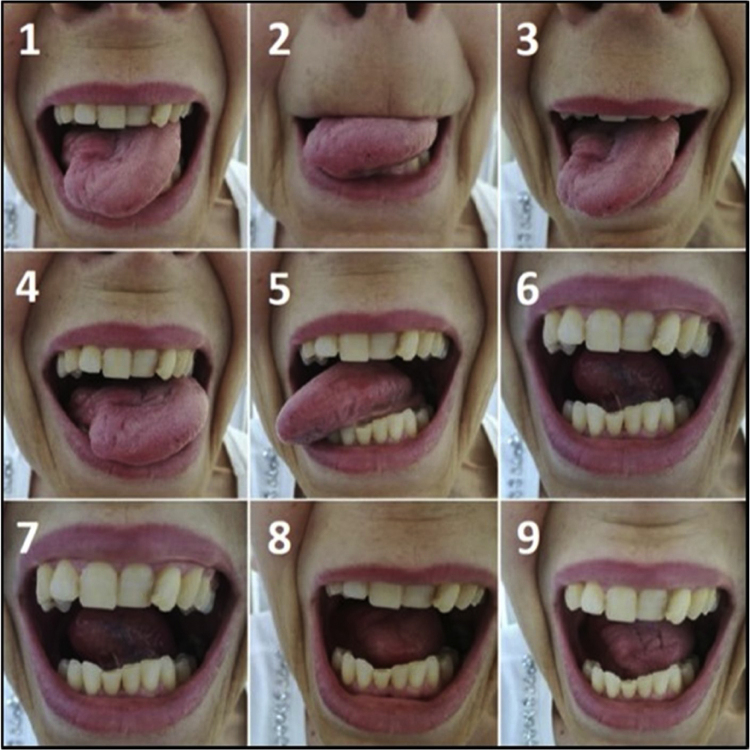
The nine movements of the tongue motility assessment in patient who underwent right partial glossectomy with pedicled flap reconstruction (platysma myocutaneous flap).

Tongue motility was better in patients without flap: mean TMA score was 2.85 ± 0.23 and 1.63 ± 0.64 in group A and B, respectively (*p* < 0.001). A statistically significant difference between the two groups was observed for every tongue movement (Figure 3, Figure 4).

**Figure 5:**
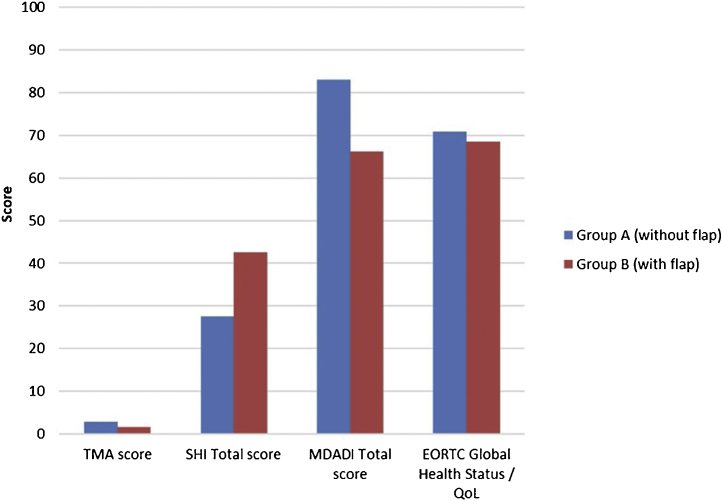
Main results (TMA score, SHI total score, MDADI total score, EORTC Global Health Status/QoL) according to reconstruction strategy.

Speech intelligibility and articulation scores did not demonstrate a significant difference between the groups (*p* > 0.05). However, some patients with greater impairment (score 3 or 4) were observed in group B (Table 3). SHI and MDADI scores were not statistically different (*p* > 0.05), but better results (lower SHI total score and higher MDADI total score) were present in group A (Table 4). QoL scores obtained with EORTC QLQ-C30 and H&N35 questionnaires were similar in the two groups (*p* > 0.05) (Table 5). Fig. 5 highlights main results according to reconstruction strategy.

## Discussion

In the last decades, the assessment of functional outcomes after oral cancer surgery has gained an important role, since achieving a good quality of life has become an essential part of the treatment.^2^ Main functions of the oral cavity are swallowing and speech articulation. Therefore, resection surgery of the tongue and surrounding tissues determines an impairment of these functions.^3^ Some parameters affect functional outcomes, including extension and localization of tongue resection, type of reconstruction, motility of residual tongue, and adjuvant RT.^3^, ^4^, ^5^, ^6^

Takatsu et al. observed that speech function was related to location and extension of tongue resection, suggesting the distinctive function of each tongue portion for the articulation of speech sounds.^10^ Bressmann et al. developed a tongue motility assessment to analyze its relationship with consonant intelligibility after partial glossectomy, showing that a higher tongue motility was related to better articulation.^4^

Small glossectomy defects may be effectively managed by primary closure, secondary intention healing, or skin or biologic grafting.^22^ Larger volume glossectomies involving at least half of the mobile tongue require flap reconstruction to re-establish bulk and shape of the tongue and to preserve the motility of the remaining tissues.^7^ Only a few contrasting data are present in the literature about intermediate volume glossectomies, analyzing the impact of reconstruction strategy on functional outcomes.^8^, ^9^, ^10^, ^11^ Indeed, it is not completely clear if flap reconstruction favors swallowing and speech in such cases. The role of flaps on tongue function is probably due to its impact on tongue motility.^4^ Common recommendations on reconstructive strategy in T2 tongue tumors are not present in the literature. Moreover, previous studies did not have homogeneous samples, since they included different volume glossectomies (from partial to total).^8^, ^9^, ^10^, ^11^ Our study aimed to analyze long-term functional outcomes in a homogeneous sample of pT2 tumors with a maximum dimension between 2 and 3 cm, after a partial glossectomy with a resection volume more than one quarter and less than half of the mobile tongue, in order to achieve more reliable results. In particular, we evaluated the role of post-operative tongue motility and its impact on swallowing and speech.

A study by Hsiao et al. compared primary closure and radial forearm flap reconstruction in 12 patients who underwent hemiglossectomy for T2–T3 carcinomas, with a followup of 6–16 months. Primary closure guaranteed better speech intelligibility and articulation, while swallowing (bolus volume and ingestion rate) was better in those with flap reconstruction.^8^ This study suggested that the bulk added by the flap improved pharyngeal clearance by maintaining the tongue-to-mouth roof contact, which is important for swallowing. On the other hand, the nonfunctional flap reduced residual tongue motility worsening articulation. Speech intelligibility was better after primary closure also in a study by Chuanjun et al.^9^ They evaluated 19 patients with T1–T2 tumors who underwent partial glossectomy or hemiglossectomy 6-months before. Radial forearm flaps or pedicled flaps were used for reconstruction (8 subjects). Ji et al. showed that patients who underwent secondary intention healing had better tongue mobility, articulation, and speech intelligibility than the free flap reconstruction group in partial glossectomy cases. On the contrary, patients who had free flap reconstruction showed better tongue mobility and speech outcomes than the secondary intention group in hemiglossectomy cases. No differences in swallowing were observed between the secondary intention and flap reconstruction groups in both partial glossectomy and hemiglossectomy cases.^23^

Follow-up duration has to be taken into account. Indeed, Lee et al. showed that both articulation and swallowing were related to follow-up duration after partial glossectomy without flap reconstruction. A slight improvement of oral functions was observed after a longer follow-up.^24^ The reason was probably an adaptation of tongue motility in speech function, while the lower improvement of swallowing may be due to the constant volume of the residual tongue during follow-up. On the other hand, Joo et al. observed that volume changes of radial forearm flap after glossectomy for T2-T3 carcinomas negatively affected speech and swallowing during the first year after surgery.^25^ To avoid a bias related to follow-up duration, in our study we analyzed patients with a minimum follow-up of 12 months. Moreover, follow-up duration was similar in the two groups.

Adjuvant RT has been indicated as a negative factor for oral functions after glossectomy.^3^, ^6^, ^26^ In our study, percentages of adjuvant RT were not statistically different between the groups. Furthermore, all the patients underwent postoperative speech and swallowing rehabilitation therapy, that was identified as a favorable factor by Furia et al.^27^ Takatsu et al. demonstrated that the positive role of rehabilitation therapy was related to the improvement of tongue motility.^10^

Our study showed good functional outcomes after partial glossectomy for small pT2 carcinomas, both with self-reporting and clinician-rating scales. Concerning tongue motility, better outcomes were observed for protrusion with depression, elevation, and dorsal elevation. On the contrary, higher impairment was reported for protrusion with elevation, and protrusion with lateralization. These results were in agreement with those by Bressmann et al.^4^ Global QoL was good in all the patients. Analyzing the whole sample, subjects with a higher tongue motility had better articulation and lower dysphagia. Therefore, this suggested that a greater preservation of tongue motility had a positive role in achieving better functional outcomes after partial glossectomy. Furthermore, higher postoperative oral functions were related to a better QoL.

Adjuvant RT negatively affected tongue motility, probably due to fibrosis of the lingual musculature. However, only a non-significant trend in favor of non-irradiated subject was present for other variables, such as articulation, swallowing, and QoL. Further studies with larger samples may probably reach statistical significance. Since adjuvant RT and speech therapy may impact oral functions, we excluded patients with less than 12-month follow-up to avoid a possible adaptation one year after oncological treatment. This justified the possibility of comparing the subjects.

In our study on partial glossectomies, tongue motility was more preserved in patients who did not undergo flap reconstruction. This suggested that flap can limit the motility of the residual tongue, in agreement with data by Hsiao et al. in hemiglossectomies and by Ji et al. in partial glossectomies.^8^, ^11^ A non-significant trend concerning speech intelligibility and articulation was observed in patients without flap reconstruction. Similar and statistically significant results were reported by Chuanjun et al. in patients who had partial glossectomy or hemiglossectomy.^9^ The non-significant trend of swallowing outcomes in favor of primary closure in our case series was confirmed by a statistically significant difference analyzing CTCAE scale for dysphagia, in contrast with previous studies.^8^, ^11^ However, these papers mainly evaluated flap reconstruction after hemiglossectomies. Finally, we did not find any difference about QoL between the two reconstruction strategies.

We observed a lower mean age in the group of patients with flap repair, due to different choice of reconstruction in older subjects (a primary closure was preferred in such cases). Along with the small number of subjects, this may represent a bias of our study. However, we found better functional outcomes in patients without flap repair despite older age which could negatively affect oral functions. Future studies with larger samples should better analyze the role of age on functional outcomes after glossectomy. Another limitation of our study is represented by the free speech sample used for evaluation, that reduces the possibility of comparison. Furthermore, the sensitivity of CTCAE dysphagia scale decrease 6-months after head and neck cancer surgery.^28^ The strength of our study was the selection of a homogeneous sample of partial glossectomies for pT2 tumors with a maximum dimension between 2 and 3 cm and a depth of invasion between 5 and 10 mm. The exclusion of small tongue resections and hemiglossectomies allowed us to obtain more accurate and valid results. Further studies with larger samples are necessary to compare functional results with free flaps to other reconstruction strategies.

## Conclusions

Partial glossectomies for pT2 tumors (within a homogeneous sample of 25%–50% of mobile tongue resections) may determine impairment of tongue motility and consequently alterations of oral functions, such as speech and swallowing. A greater preservation of tongue motility has a positive role in achieving better speech articulation and swallowing. Moreover, worse functional outcomes induce a lower quality of life. The reconstruction strategy impacts long-term outcomes. In particular, avoiding pedicled flaps seems to guarantee lower impairment of speech and swallowing. Therefore, the type of reconstruction should be adequately evaluated and planned before surgery. Further studies are mandatory to better evaluate the role of age and differences between primary closure and second intention healing after partial glossectomy.

## Conflicts of interest

The authors declare no conflicts of interest.
